# p62/SQSTM1-Dependent Autophagy of Lewy Body-Like α-Synuclein Inclusions

**DOI:** 10.1371/journal.pone.0052868

**Published:** 2012-12-31

**Authors:** Yoshihisa Watanabe, Harutsugu Tatebe, Katsutoshi Taguchi, Yasuhisa Endo, Takahiko Tokuda, Toshiki Mizuno, Masanori Nakagawa, Masaki Tanaka

**Affiliations:** 1 Department of Basic Geriatrics, Graduate School of Medical Science, Kyoto Prefectural University of Medicine, Kawaramachi-Hirokoji, Kamikyo-ku, Kyoto, Japan; 2 Department of Neurology, Graduate School of Medical Science, Kyoto Prefectural University of Medicine, Kawaramachi-Hirokoji, Kamikyo-ku, Kyoto, Japan; 3 Department of Applied Biology, Kyoto Institute of Technology, Matsugasaki, Kyoto, Japan; Stanford University School of Medicine, United States of America

## Abstract

α-Synuclein is the main component of Lewy bodies, the intraneuronal inclusion bodies characteristic of Parkinson’s disease. Although α-synuclein accumulation is caused by inhibition of proteasome and autophagy-lysosome, the degradation of α-synuclein inclusions is still unknown. Formation of Lewy body-like inclusions can be replicated in cultured cells by introducing α-synuclein fibrils generated *in vitro*. We used this cell culture model to investigate the autophagy of α-synuclein inclusions and impaired mitochondria. The intracellular α-synuclein inclusions immediately underwent phosphorylation and ubiquitination. Simultaneously they were encircled by an adaptor protein p62/SQSTM1 and directed to the autophagy-lysosome pathway in HEK293 cell line. Most phospho-α-synuclein-positive inclusions were degraded in 24 h, however, lysosomal dysfunction with bafilomycin A1 significantly affected their clearance. Moreover, inhibition of autophagy by Atg-5 siRNA treatment reduced the incorporation of α-synuclein inclusions into LC3-positive autophagosomes. Knockdown experiments demonstrated the requirement of p62 for α-synuclein autophagy. These results demonstrate that α-synuclein inclusions are preferred targets for p62-dependent autophagy. Next, we investigated the autophagic clearance of impaired mitochondria in α-synuclein inclusion-containing cells. Impaired mitochondria were almost completely eliminated after mitochondrial uncoupling even in the presence of α-synuclein inclusions, suggesting that mitochondrial clearance is not prevented by α-synuclein inclusions in HEK293 cells.

## Introduction

Aggregation of α-synuclein is a common neuropathological hallmark in Parkinson’s disease (PD), dementia with Lewy bodies (DLB), and multiple systems atrophy [1]. The genetic evidence for α-synuclein in familial PD emerged from the identification of three missense mutations (A30P, E46K, and A53T) and triplications of the wild-type α-synuclein gene [2]. Although the mechanism of α-synuclein aggregation is unknown, the concentration and conformation of α-synuclein may be relevant to PD pathogenesis [3]. *In vitro* studies suggest the hypothesis that α-synuclein, an intrinsically unfolded protein, is changed to a β-sheet-rich form, which is oligomerized or fibrilized nucleation-dependently [4]. Aggregation-prone proteins are degraded by the ubiquitin-proteasome system (UPS) and the autophagy–lysosome pathway [5]. Indeed, α-synuclein is ubiquitinated and degraded by both the UPS and autophagy [6], [7], [8], suggesting that these two systems are involved in α-synuclein homeostasis. Although both chaperone-mediated autophagy (CMA) and macroautophagy pathway are important in its autophagic degradation [6], [9], [10], it has been unknown which species of α-synuclein including monomer, oligomer, or inclusion form are preferred targets for them. Recent findings have supported the notion that impairment of the autophagy pathway is related to the development of PD [10], [11], [12]. Mutant α-synuclein inhibited autophagy by tightly binding to the receptor on the lysosomal membrane [10]. Furthermore, parkin and PTEN-induced kinase 1 (PINK1), other familial PD-associated gene products, are required for clearance of damaged mitochondria by selective autophagy [11], [12]. However, interplay between α-synuclein and parkin/PINK1in autophagy has not yet been demonstrated.

Although α-synuclein inclusion formation in cultured cells has repeatedly been reported, these models are limited in providing abundant α-synuclein inclusions that display the major features of Lewy bodies [13]. On the other hand, it is possible to form α-synuclein fibrils *in vitro*, which have similar appearance to Lewy body filaments [14]. Interestingly, when α-synuclein fibrils generated *in vitro* were introduced into cells using calcium phosphate precipitation, cationic liposomes, or a transfection reagent, Lewy body-like inclusions containing phosphorylated and ubiquitinated α-synuclein were readily detectable in these cells [13], [15], [16]. This exogenic fibril-introduction system may be used to recapitulate Lewy body formation in cultured cells. In this study, we investigated autophagic clearance of Lewy body-like α-synuclein inclusions and their influence on mitophagy using this cell culture model. We show that α-synuclein inclusions are preferred targets for p62-dependent autophagy.

## Results

**Figure 1:**
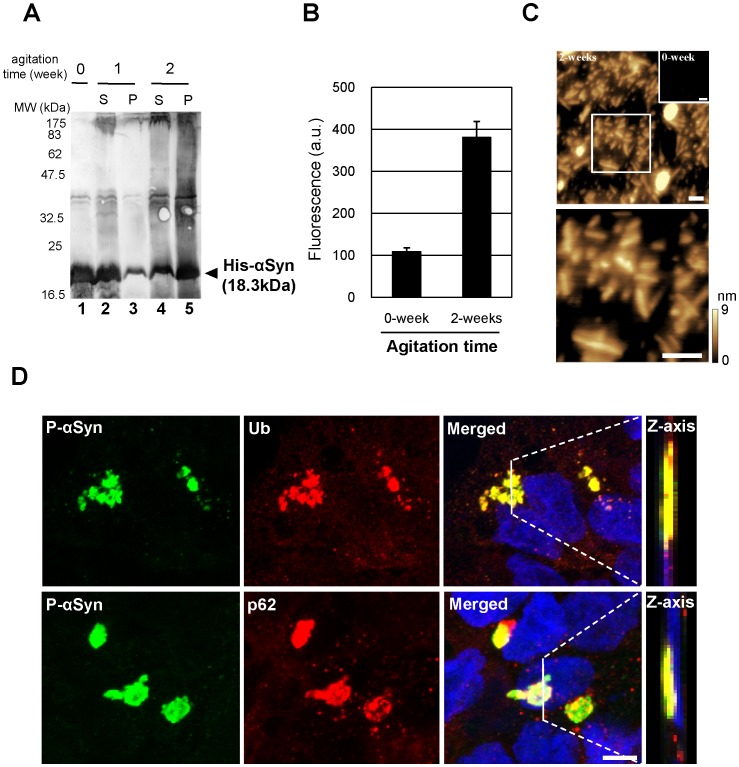
Formation of Lewy body-like α-synuclein inclusions in cultured cells. (**A**) Purified His-α-synuclein protein was agitated for 0–2 weeks (lane 1, 0weeks; lanes 2 and 3, 1week; lanes 4 and 5, 2weeks). Aliquots from each sample were separated into supernatant (lanes 2 and 4) and pellet (lanes3 and 5) fractions. These fractions were subjected to immunoblotting with anti-α-synuclein antibody. Numbers at left are molecular weight markers (MW) in kilodaltons. (**B**) Fibril formation of α-synuclein was measured with ProteoStat, an aggregation detection dye. a.u., arbitrary units. (**C**) AFM images of α-synuclein agitated for 0 or 2 weeks were acquired in air using the dynamic mode. A high-magnification view of the boxed area is shown in the lower panel. The scale on the right represents the height of pixels in the image. Scale bars, 100 nm. (**D**) α-Synuclein inclusions in fibril-introduced HEK293 cells were detected immunocytochemically with anti-phosphorylated α-synuclein (P-αSyn), anti-ubiquitin (Ub), and anti-p62 (p62) antibodies. Confocal images reconstructed in the z-axis along the white lines are shown in the right panel (Z-axis). Blue, DAPI. Scale bar, 10 µm.

It was recently reported that Lewy body-like inclusions including phosphorylated α-synuclein were observed in cultured cells when α-synuclein fibrils generated *in vitro* were introduced by transfection [13], [15], [16]. We purified His-α-synuclein recombinant protein from *E. coli* and prepared α-synuclein fibrils by agitation for 2 weeks (Fig. 1A). Although the majority of His-α-synuclein agitated for 1 week was recovered in the supernatant, more than 75% of this protein moved to the pellet fraction after 2 weeks of agitation, as assessed by band densitometry (Fig. 1A). His-α-synuclein was detected as an 18.3-kDa band and a high-molecular-weight smear by western blot analysis (Fig. 1A, lane 5), suggesting that these fibrils contained both SDS-soluble and -insoluble forms. Moreover, we quantified and observed α-synuclein fibril formation. After 2 weeks’ agitation, α-synuclein fibrils were obviously detected with ProteoStat detection dye (Fig. 1B), and atomic force microscope (AFM) demonstrated that α-synuclein fibrils existed in the agitated sample (Fig. 1C). The fibrils were 5–10 nm high and 30–100 nm long.

We then transfected the α-synuclein fibrils into HEK293 cells using Lipofectamine LTX reagent. We confirmed α-synuclein inclusion formation in these cells immunocytochemically using anti-phospho α-synuclein monoclonal (Fig. 1D). Phosphorylated α-synuclein inclusions (1–4 µm diameter) were specifically detected in the cytoplasm when α-synuclein fibrils were introduced into cells (Fig. 1D, P-αSyn). One hour after fibril-introduction, phosphorylated α-synuclein-positive cells were about 7%. Considering the fibril size, a large number of the introduced fibrils must have assembled into α-synuclein inclusions. As shown in Fig. 1D, these inclusions underwent several modifications such as phosphorylation and ubiquitination. It is well known that p62/SQSTM1, an adaptor protein, is a component of Lewy bodies, neurofibrillary tangles, and Mallory bodies [17]. We therefore examined whether p62 co-localized with the α-synuclein inclusions generated in cultured cells. p62 apparently accumulated at phospho-α-synuclein-positive inclusions (Fig. 1D, p62). According to previous result [16], almost every inclusion was colocalized with ubiquitin and p62. In contrast, these inclusions were never detected when monomeric α-synuclein was introduced in cells (Fig. S1). Our finding that the α-synuclein inclusions were phosphorylation-, ubiquitin-, and p62-positive matches the features of Lewy bodies.

**Figure 2:**
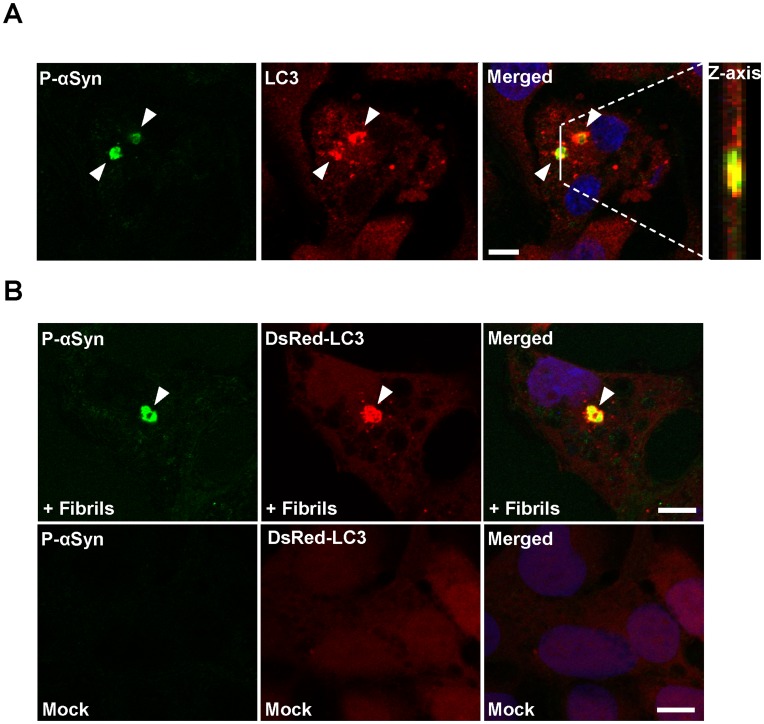
Autophagic clearance of α-synuclein inclusions. (**A**) Autophagic clearance of α-synuclein inclusions was verified by immunocytochemical analysis with anti-phosphorylated α-synuclein (P-αSyn) and anti-LC3 (LC3) antibodies. Confocal images reconstructed in the z-axis along the white lines are shown in the right panel (Z-axis). Blue, DAPI. Scale bar, 10 µm. Phosphorylated α-synuclein-positive inclusions were sequestrated into LC3-positive autophagosomes. (**B**) Similarly, this sequestration was confirmed in HEK293 cells stably expressing DsRed-LC3. The upper and lower panels show α-synuclein fibrils-and mock-introduced cells, respectively. Blue, DAPI. Scale bar, 10 µm.

**Figure 3:**
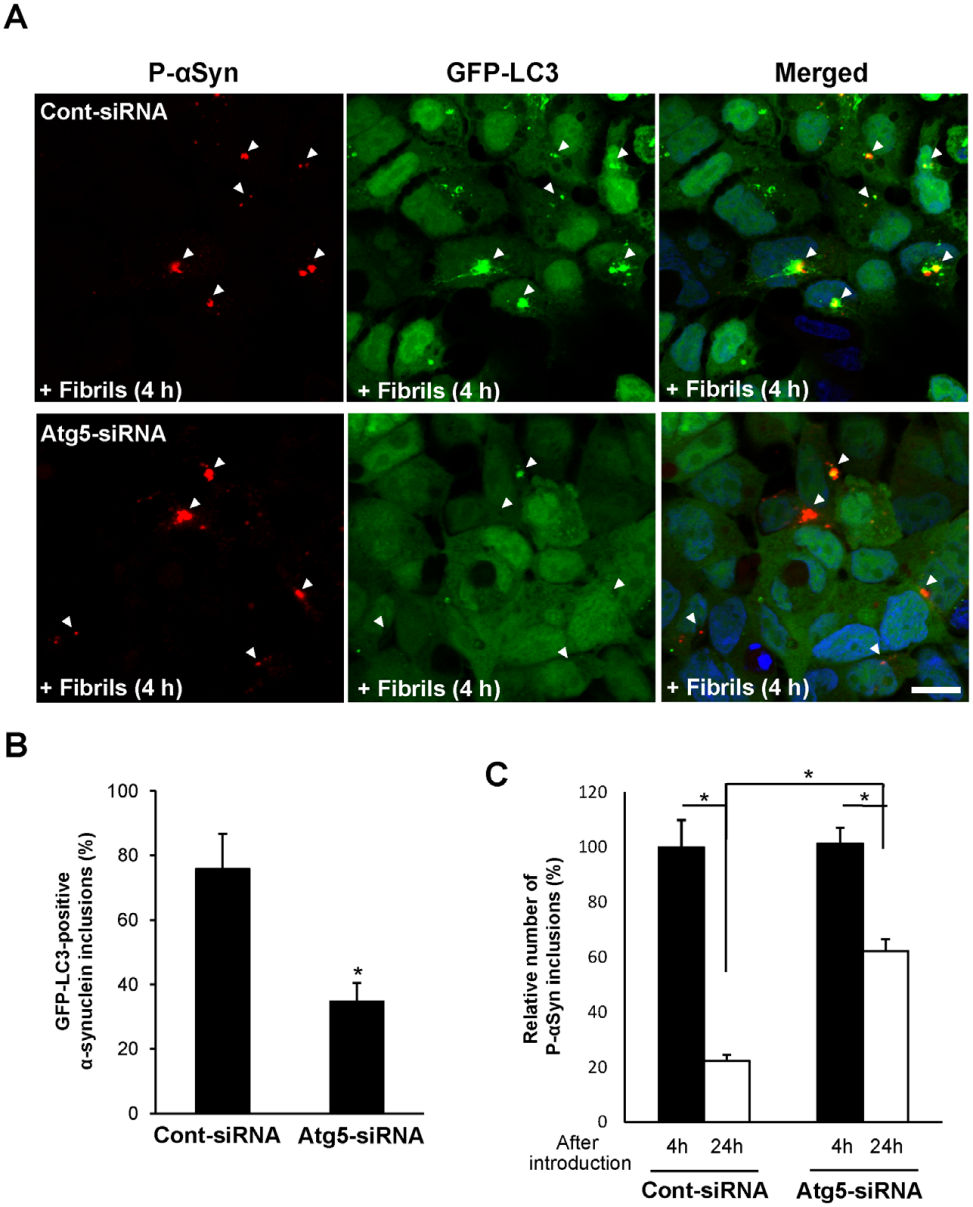
The reduced incorporation of α-synuclein inclusions into autophagosomes by autophagy inhibition. (**A**) HEK293 cells stably expressing GFP-LC3 were transfected with control (upper) or Atg-5 siRNA (lower). After 36 h, α-synuclein fibrils were introduced into them for 4 h, followed by immunostaining with anti-phospho-α-synuclein antibody (P-αSyn). Blue, DAPI. Scale bar, 10 µm. (**B**) The number of GFP-LC3-positive α-synuclein inclusions (%) were assessed in randomly chosen fields (n = 5) with 48–141 inclusions. Statistical analysis was performed with *t*-test (*p<0.01). (**C**) Clearance of α-synuclein inclusions in control (solid bar) or Atg-5 (open bar) knockdown cells was measured at 4 h and 24 h after α-synuclein fibrils. The number of phosphorylated α-synuclein-positive cells was assessed in randomly chosen fields (n = 5). Data from each experiment are were normalized to Cont-siRNA-4 h (100%) and represented as relative value of α-synuclein inclusions ± s.e.m. Statistical analysis was performed with one-way ANOVA (post-hoc Tukey’s test). *p<0.01.

Using our cell culture model, we investigated whether α-synuclein inclusions were degraded by autophagy, because α-synuclein oligomeric intermediates are a known target for autophagy [18]. α-Synuclein fibrils were introduced into HEK293 cells expressing a marker of autophagosomes, GFP-LC3. One hour after transfection, GFP-LC3 dot structures were observed in the cytoplasm (Fig. S2, arrowheads). Moreover, sequestration of α-synuclein inclusions into autophagosomes was demonstrated by immunocytochemistry. Phosphorylated α-synuclein-positive inclusions were encircled by endogenous LC3 protein or DsRed-LC3 protein in HEK293 cells (Fig. 2A and B, arrowheads). About 60% of α-synuclein inclusions were sequestrated into LC3. Similarly, colocalization patterns of LC3, p62, or ubiquitin with phosphorylated α-synuclein-positive inclusions were detected in neuroblastoma SH-SY5Y cells (Fig. S3). However, it was reported that LC3 could be autophagy-independently localized with p62-positive aggregates [19]. To examine whether the incorporation of α-synuclein inclusions into LC3 was attributed to autophagy, we performed the *atg*-5 (autophagy associated gene 5) knockdown experiment. α-Synuclein fibrils were introduced into HEK293 cells stably expressing GFP-LC3 for 4 h, following autophagy suppression by treatment with Atg-5 siRNA. GFP-LC3 was localized to phosphorylated α-synuclein inclusions in the case of control siRNA treatment (Fig. 3A, upper panels). In contrast, Atg-5 siRNA treatment reduced their incorporation into GFP-LC3 dots (Fig. 3A, lower panels). The number of GFP-LC3–positive α-synuclein inclusions was significantly decreased in autophagy-suppressed cells (Fig. 3B). In Atg-5 knockdown cells, the degradation of α-synuclein inclusions was significantly reduced 24 h after fibril introduction (Fig. 3C and S4), suggesting that the colocalization of LC3 with α-synuclein inclusions was dependent on autophagy.

**Figure 4:**
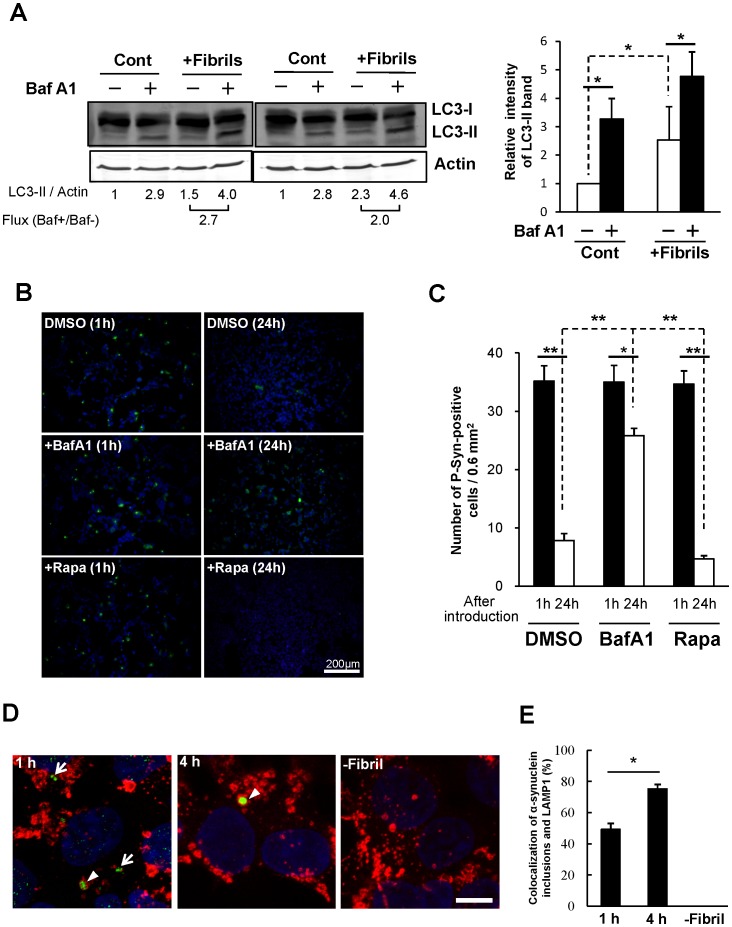
Autophagic clearance of α-synuclein inclusions. (**A**) Autophagy flux was analyzed by LC3 protein monitoring. α-Synuclein fibrils-introduced HEK293 cells were treated with (+)/without (−) bafilomycin A1 (BafA1) for 2 h. Cell lysates were subjected to immunoblotting analysis using anti-LC3 (upper panel) or anti-actin (lower panel) antibodies. Quantification of the relative levels of LC3-II/Actin or autophagic flux (Baf+/Baf-) is shown with the ratio. Relative level of LC3-II/Actin was represented by mean ± s.d. as a graph. Statistical analysis was performed with one-way ANOVA (post-hoc Tukey’s test). *p<0.05. This experiment was repeated three times. (**B**) α-Synuclein fibrils were introduced into HEK293 cells, followed by addition of DMSO, 100 nM bafilomycin (BafA1), or 200 nM rapamycin (Rapa). Cells were stained by anti-phosphorylated α-synuclein antibody (green) and DAPI (blue) 1 h (left panels) or 24 h (right panels) after introduction. (**C**) The number of phosphorylated α-synuclein-positive cells per unit area (0.6 mm^2^) was assessed in randomly chosen fields (n = 6). Data represent mean ± s.e.m. Statistical analysis was performed with one-way ANOVA (post-hoc Tukey’s test). *p<0.05, **p<0.001. (**D**) Colocalization of α-synuclein inclusions and lysosomes. α-Synuclein inclusions (P-αSyn) and lysosomes (LAMP1) were immunostained with anti-phospho-α-synuclein polyclonal antibody (green) and anti-LAMP1 antibody (red) 1 h (left panel) or 4 h (middle panel) after fibril introduction. Fibril-unintroduced cells were used as mock control (right panel). Arrowheads and arrows indicate localized or unlocalized α-synuclein inclusions to lysosomes, respectively. Blue, DAPI. Scale bars, 10 µm. (**E**) Colocalization of α-synuclein inclusions (n = 41–73) to lysosomes were measured in randomly chosen fields. The experiment was repeated three times. Data represent mean ± s.e.m. Statistical analysis was performed with one-way ANOVA (post-hoc Tukey’s test). *p<0.01.

Furthermore, we verified whether autophagy activity was induced by the accumulation of these inclusions. The relative intensity of LC3-II band, which was C-terminally conjugated to phosphatidylethanolamine, was significantly increased after introduction of α-synuclein fibrils (Fig. 4A). The presence of autophagic flux was also confirmed using bafilomycin A1 (Baf A1), a specific inhibitor of V-ATPase. The autophagy flux was calculated as the ratio between LC3-II with BafA1 and without BafA1 (Fig. 4A; Flux), which was an average value of 2.1 in fibrils-introduced cells. This result indicated that autophagy was induced by the appearance of α-synuclein inclusions. As shown in Fig. 4B and 4C, phospho-α-synuclein-positive inclusions were formed by 1 h after fibril-introduction and were mostly degraded after 24 h (DMSO). Their degradation was accelerated by the treatment with the autophagy inducer rapamycin (Fig. 4B and 4C; Rapa). However, inhibition of lysosomal function with bafilomycin A1 significantly affected their clearance (Fig. 4B and 4C; BafA1). Furthermore, α-synuclein inclusions were colocalized with LAMP1, lysosomal associated membrane protein-1 (Fig. 4D). LAMP1-positive α-synuclein inclusion was detected in about 50% of inclusions one hour later (Fig. 4E, 1 h). Four hours after fibril-introduction, there was a further increase in this colocalization (Fig. 4E, 4 h; 75% of inclusions). This indicates that α-synuclein inclusions are targets for autophagic clearance.

**Figure 5:**
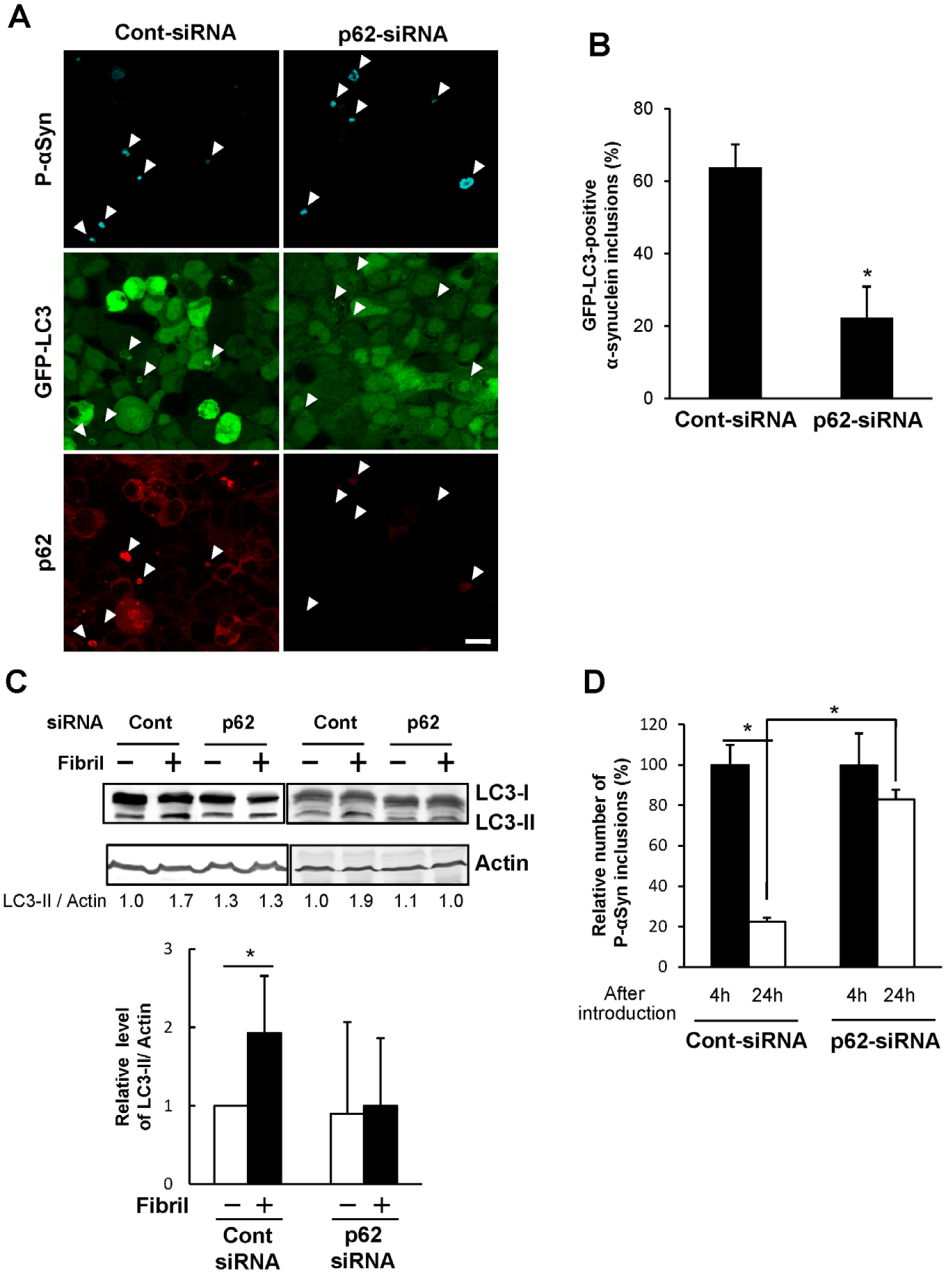
Involvement of p62 in the selective clearance of α-synuclein inclusions. (**A**) HEK293 cells stably expressing GFP-LC3 were transfected with p62-siRNA (right panels) or control-siRNA (left panels). Phosphorylated α-synuclein-positive inclusions (P-αSyn) are indicated by arrowheads. In p62-knockdown cells, the expression and accumulation of p62 were not detected, and inclusions were never sequestrated by GFP-LC3 autophagosomes. Scale bars, 20 µm. (**B**) The number of GFP-LC3-positive α-synuclein inclusions (%) in Fig. 5A were assessed in randomly chosen fields with 32–44 inclusions (n = 3). Data represent mean ± s.e.m. Statistical analysis was performed with *t*-test (*p<0.05). (**C**) α-Synuclein fibrils were introduced into control (Cont) or p62-knockdown (p62) cells. Four hours after introduction, cell lysates were subjected to immunoblotting analysis using anti-LC3 (upper panel) or anti-actin (lower panel) antibodies. Quantification of the relative levels of LC3-II/Actin is shown with the ratio. Relative level of LC3-II/Actin was represented by mean ± s.d. as a graph. Statistical analysis was performed with one-way ANOVA (post-hoc Tukey’s test). *p<0.01. This experiment was repeated three times. (**D**) Clearance of α-synuclein inclusions in control (solid bar) or p62 (open bar) knockdown cells was measured at 4 h and 24 h after α-synuclein fibrils. The number of phosphorylated α-synuclein-positive cells was assessed in randomly chosen fields (n = 5). Data from each experiment were normalized to Cont-siRNA-4 h (100%) and represented as relative value of α-synuclein inclusions ± s.e.m. Statistical analysis was performed with one-way ANOVA (post-hoc Tukey’s test). *p<0.01.

Recently, it has been reported that p62 is involved in selective autophagic clearance of multiple substrates such as protein aggregates, damaged organelles, and invasive microorganisms [20], [21], [22]. α-Synuclein inclusions would be expected to undergo selective autophagy because p62 colocalized to these inclusions, as shown in Fig. 1. We then examined the autophagic clearance of inclusions in p62-knockdown cells (Fig. 5). Although α-synuclein inclusions formed normally in p62-knockdown cells, they were not encircled by GFP-LC3 (Fig. 5A). We quantified the numbers of GFP-LC3 positive α-synuclein inclusions in these cells. More than 60% of α-synuclein inclusions were encircled by GFP-LC3 in cells transfected with control siRNA (Fig. 5B, Cont-siRNA), whereas GFP-LC3 positive inclusions were significantly decreased by p62-knockdown (Fig. 5B, p62-siRNA). This finding was confirmed by western blot analysis of endogenous LC3 (Fig. 5C). The relative intensity of LC3-II band was significantly increased 2.0-fold in control cells, whereas there was no increase in its intensity in p62 knockdown cells (Fig. 5C). Moreover, the degradation of α-synuclein inclusions was significantly reduced in p62 knockdown cells as well as in Atg-5 knockdown cells (Fig. 5D and S4). Therefore, α-synuclein inclusions were considered to be degraded by autophagy in a p62-dependent manner.

**Figure 6:**
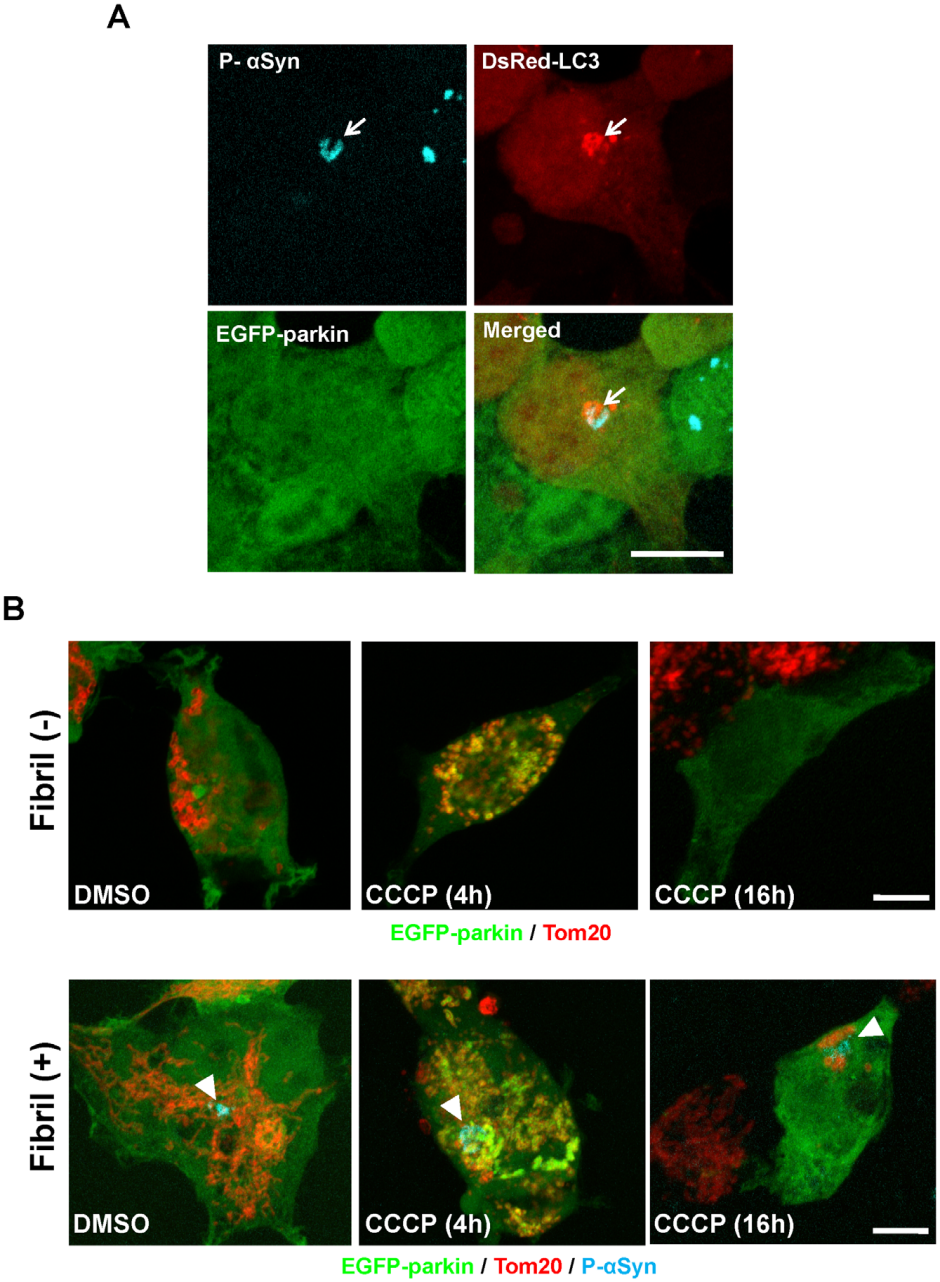
Impairment of mitochondria and mitophagy in cells harboring α-synuclein inclusions. (**A**) α-Synuclein fibrils were introduced into HEK293 cells stably expressing both DsRed-LC3 and EGFP-parkin. After 24 h, the cells were immunostained with anti-phosphorylated α-synuclein (P-αSyn). There was no alteration in the localization of EGFP-parkin. The arrows indicate α-synuclein inclusion. Scale bar, 10 µm. (**B**) Mitochondrial clearance was confirmed in mock- (upper panels) or α-synuclein fibrils-introduced cells (lower panels). After introduction, EGFP-parkin cells were treated with DMSO (left) or 10 µM CCCP for 4 h (middle) or 16 h (right). Cells were examined immunocytochemically with anti-Tom20 (red) and anti-phosphorylated α-synuclein antibody (P-αSyn; aqua). Figures were presented as merged images. Individual images were provided in supplemental figure (Fig. S8). Scale bar, 10 µm.

Next, we examined whether mitochondrial function or autophagic clearance of damaged mitochondria was affected by α-synuclein inclusions. HEK293 cells stably expressing EGFP-parkin and DsRed-LC3 were generated to monitor mitochondrial dysfunction and mitophagy, which could reproduce recruitment of EGFP-parkin to impaired mitochondria and autophagy induction upon carbonyl cyanide m-chlorophenylhydrazone (CCCP) treatment to uncouple the mitochondria for 4 h (Fig. S5). α-Synuclein fibrils were introduced into EGFP-parkin/DsRed-LC3-expressing cells, followed by incubation for 24 h. EGFP-parkin exhibited a uniform distribution even in the presence of phosphorylated α-synuclein-positive inclusions, which were sequestrated into LC3-positive autophagosomes (Fig. 6A). Next, these cells were treated with 10 µM CCCP for 4 h (Fig. S6). EGFP-parkin was recruited to damaged mitochondria even in inclusion-positive cells, and DsRed-LC3 puncta were also localized in the close vicinity of damaged mitochondria (Fig. S6, arrowheads). Furthermore, mitochondrial clearance was confirmed by the presence of the mitochondrial marker Tom20 after 16 h of CCCP treatment (Fig. 6B). In agreement with previous reports [11], [23], the effective clearance of impaired mitochondria after CCCP treatment was never observed in HEK293 cells (Fig. S7). However, EGFP-parkin overexpression induced the degradation of impaired mitochondria, resulting in their disappearance after 16 h (Fig. 6B and S8A). As well as in the α-synuclein inclusion-negative cells, their clearance was also observed in the EGFP-parkin expression cells accumulating α-synuclein inclusion (Fig. 6B and S8B). There was no major difference in the mitochondrial elimination-rate between α-synuclein inclusion-negative and –positive cells. Thus, the existence of α-synuclein inclusions dose not seem to affect mitochondrial function or autophagic clearance of impaired mitochondria in HEK293 cells.

## Discussion

Most aggregation-prone proteins are degraded by the UPS and/or autophagy pathways. α-Synuclein is also cleared by these proteolytic systems, supporting the hypothesis that impaired proteasomal and autophagic degradation of α-synuclein is an important mechanism of neurodegeneration in PD [6], [7], [8], [10]. Lee et al. reported that α-synuclein oligomeric intermediates, rather than fibrillar inclusion bodies, are targets for the lysosomal degradation pathway including autophagy [18]. In medaka fish or the SH-SY5Y human dopaminergic cell line, the treatment with ammonium chloride, a lysosome inhibitor, induces the formation of LC3-positive α-synuclein inclusions [24]. Although these reports suggest that autophagy-lysosome pathway is important in α-synuclein degradation, it has not been elucidated whether α-synuclein inclusions are also targets for this pathway. Our cell culture model demonstrated that α-synuclein inclusions were sequestrated into autophagosomes and colocalized to lysosomes immediately after introducing α-synuclein fibrils. The fact that autophagosomes are localized to Lewy bodies in the brains of patients with DLB [25] suggests that not only α-synuclein oligomers but also inclusions are preferred targets for autophagy. Furthermore, these inclusions were ubiquitinated and encircled by the p62 adaptor protein, consistent with the pathological features of Lewy bodies and Lewy neuritis [17], [26]. p62 is involved in inclusion formation and selective autophagy of several ubiquitinated substrates [21], [22], [27]. Our *p62*-knockdown experiment demonstrated that p62 is required for autophagic clearance of α-synuclein inclusions but not for inclusion formation from introduced α-synuclein fibrils. Aggregation analysis in p62 or histone deacetylase 6 (HDAC6) deficient cells suggests that p62 may have a role in early stage from soluble form to microaggregate, whereas HDAC6 transports protein aggregates to the aggresome-like inclusion bodies downstream of p62 [28]. In our experiments, fibrous α-synuclein agitated *in vitro* is introduced in cells. Hence, p62 might be no longer necessary for inclusion formation of it in the cytoplasm because prepared α-synuclein already forms microaggregates.

Our study provides evidence for autophagic clearance of α-synuclein inclusions. Although UPS are also involved in α-synuclein metabolism [8], [29], it is uncertain whether α-synuclein inclusions are preferred targets for it. However, our result demonstrates that UPS might be involved in degradation of soluble or small α-synuclein aggregates but not large inclusions because degradation of α-synuclein inclusions is suppressed by lysosomal inhibition. Indeed, the UPS is the main degradation pathway for α-synuclein under normal conditions *in vivo* while with increased α-synuclein burden the ALP is recruited, suggesting that UPS and autophagy have distinct roles in degradation of α-synuclein [6].

Functional analysis of parkin and PINK1 has revealed that impaired mitochondria are eliminated through autophagy, and their accumulation contributes to PD pathogenesis [30]. α-Synuclein has been reported not only to be targeted for autophagy but also to have an influence on autophagy or mitophagy, for example, autophagy impairment by α-synuclein overexpression [31] or induction of mitophagy and neuronal cell death by A53T mutant α-synuclein overexpression [32]. In these experiments, however, it has remained undefined the relationship between autophagy and α-synuclein inclusion, one of the pathological hallmarks of PD. Recently, Tanji et al. revealed that Atg-8 homologues, LC3 and γ-aminobutyric-acid type A receptor associated proteins (GABARAPs), were localized in Lewy bodies in PD and DLB, suggesting that autophagic function is impaired through alteration of Atg8 homologues in Lewy body disease [33]. We assumed that autophagic clearance of impaired mitochondria would be competitively prevented by α-synuclein inclusions. However, damaged mitochondria seemed to be normally degraded by autophagy in our cell culture model since impaired mitochondria were almost completely eliminated after 16 h of CCCP treatment even in the presence of α-synuclein inclusions. Although this result indicates that mitophagy would not be prevented by the accumulation of α-synuclein inclusions in HEK293 cells, it is unknown whether the aging neurons possess the same level of autophagic activity. Indeed, genome-wide analysis revealed that several key players in the autophagic pathway, such as *atg*-5 and *atg*-7, show decreased expression in the aging brains [34]. Moreover, toxic oligomeric α-synuclein but not inclusions may influence the accumulation of impaired mitochondria or a dysfunction of autophagy/mitophagy. Considering these, our results need to be further verified in the aging neurons or α-synuclein oligomer-introduced cells.

## Materials and Methods

### Cells

All cells were grown in Ham’s F12 medium supplemented with 10% fetal bovine serum (FBS). Flp-In T-REx HEK293 cells stably expressing DsRed-LC3 or GFP-LC3 were established as described previously [35]. Expression of DsRed-LC3 or GFP-LC3 protein was induced with 1 µg/ml tetracycline. Cells were treated with 100 nM bafilomycin A1 (Wako Pure Chemical Industries, Osaka, Japan), 200 nM rapamycin (Alexis Biochemicals, San Diego, CA), or 10 µM CCCP (Sigma-Aldrich, St Louis, MO).

### Antibodies

Anti-α-synuclein antibody (Syn-1), anti-phospho-α-synuclein antibody (Ser129), and anti-Tom20 antibody (FL-145) were purchased from BD Biosciences (San Diego, CA), Wako Pure Chemical Industries, and Santa Cruz Biotechnology (Santa Cruz, CA), respectively. The anti-ubiquitin (FL-76; Santa Cruz Biotechnology), anti-LC3 antibody, and anti-p62 antibody were used as described previously [35]. In part, phosphorylated α-synuclein was detected with anti-α-synuclein (phosphor S129) rabbit polyclonal antibody (Abcam, Cambridge, MA).

### Plasmid Construction and Production of Stable Cell Lines

A pEGFP-parkin plasmid was constructed by PCR using the primers 5′-AAGCTTTGATAGTGTTTGTCAGGTTCAAC-3′ and 5′-GGATCCTACACGTCGAACCAGTGGTC-3′. The PCR product was inserted into the *Hind*III–*BamH*I sites of pEGFP-C1. The plasmid was stably transfected into Flp-In T-REx HEK293 DsRed-LC3 cells. Cells were passaged into medium containing G418 (200 µg/ml) for 10 days, followed by colony isolation using a paper disc (Thermo Fisher Scientific, Fremont, CA).

### Introduction of α-synuclein Fibrils

Recombinant His-α-synuclein was purified from *E. coli*, as described previously [36]. Bacterially expressed His-α-synuclein was resuspended in phosphate-buffered saline containing 0.5% Triton X-100 and 1 mM phenylmethylsulfonyl fluoride and extracted by sonication. Extracts were heat-denatured at 100°C for 10 min, followed by centrifugation at 14,000 × *g* for 20 min. The clear lysate was applied to Ni-chelating resin (Nacalai Tesque, Kyoto, Japan), and His-α-synuclein was purified by washing with 20 mM imidazole and elution with 300 mM imidazole. Subsequently, the effluent was purified by gel filtration chromatography (Superose6; GE Healthcare, Little Chalfont, UK). *In vitro* fibril formation of His-α-synuclein was carried out in 20 mM Tris-Cl (pH 7.5) buffer by agitation (500 rpm) at 25°C for 2 weeks, and fibrils were recovered by ultracentrifugation (110,000×*g*, 20 min). Fibril formation was monitored with a ProteoStat Protein Aggregation Assay kit (ENZO LIFE SCIENCES, Plymouth Meeting, PA), according to the manufacturer’s instructions. The ProteoStat Detection Reagent Loading Solution was mixed with 1 mg/ml of protein sample, and the fluorescence intensity was measured with a CytoFluor 2300 (excitation at 530 nm/emission at 590 nm; Applied Biosystems, Foster City, CA). His-α-synuclein fibrils (200 µg/ml) were incubated with 1.25 µl of Lipofectamine LTX (Invitrogen) and CombiMag (OZ Biosciences, Marseille, France) for 45 min, and these mixtures were added to FBS-free culture medium at a final concentration of 4 µg/ml, before introduction of protein upon the magnetic plate for 30 min. After introduction, cells were cultured in Ham’s F12 medium supplemented with 10% FBS.

### Atomic Force Microscopy (AFM)

The agitated sample was placed on freshly cleaved mica (NILACO, Tokyo, Japan) and incubated for 2 min. Images were obtained with an SPM-9700 (SHIMADZU, Kyoto, Japan) operating in Dynamic Mode. The scanning parameters were: free oscillation amplitude, 0.3 V; operating point, 0.175–0.196 V; drive (tapping) frequency, 276 kHz; and scan rate, 1.0 Hz.

### Immunocytochemistry and Confocal Microscopy

Immunocytochemical analysis was performed as described previously [35]. Cells were fixed with 4% paraformaldehyde after the various drug treatments. For immunostaining with anti-LAMP1 monoclonal antibody (H4A3), cells were fixed with 4% paraformaldehyde. The fixed cells were permeabilized in phosphate-buffered saline (PBS) containing 0.1% Triton X-100 and blocked with blocking solution (Nacalai Tesque). The cells were then incubated with anti-phospho-α-synuclein, anti-ubiquitin, anti-LC3, or anti-p62 antibodies (1∶500 dilution) for 12 h at 25°C. After three washes with PBS, the cells were incubated with Fluorescein-labeled anti-mouse IgG (Vector Laboratories, Burlingame, CA), Alexa Fluor 555-labeled goat anti-mouse IgG (Invitrogen), Cy3-labeled goat anti-rabbit IgG (Millipore, Billerica, MA), Alexa Fluor 688-labeled goat anti-rabbit IgG (Invitrogen), or Alexa Fluor 350-labeled goat anti-mouse IgG (Invitrogen) for 4 h at 25°C. After washing and staining with 4',6-diamidino-2-phenylindole (DAPI; Dojindo, Kumamoto, Japan), the cells on the cover glasses were mounted on glass slides in an aqueous mounting medium (FluorSave Reagent; Calbiochem, Darmstadt, Germany) and examined under a fluorescence microscope (Olympus, Tokyo, Japan) or a confocal laser microscope (LSM 510 META; Carl Zeiss, Oberkochen, Germany). Confocal laser-scanned images were obtained with an LSM 510 META (Carl Zeiss, Oberkochen, Germany). EGFP and Fluorescein were excited using a 488-nm argon laser, and emission was recorded through a BP 500–530-nm filter. DsRed-monomer and Cy3 was excited with a 543-nm helium-neon laser, and emission was recorded through an LP 560-nm filter. DAPI or Alexa Fluor 350 was excited at 800 or 780 nm, respectively, with a Mai Tai two-photon laser, and fluorescence was recorded through a 390–465 nm BP filter. Images were obtained with a Plan-Apochromat 100×/1.4 Oil DIC lens. Lysosomes were visualized with anti-LAMP1 antibody.

### Autophagy Flux Analysis

α-Synuclein fibrils-introduced HEK293 cells were treated with/without 100 nM Bafilomycin A1 for 2 h. Cells were washed with PBS and lysed with 1×Laemmli sample buffer. Cell lysates were subjected to immunoblotting analysis with anti-LC3 or anti-actin antibody. The relative intensity of the LC3-II band was determined sing Image J software.

### RNA Interference

Knockdown of *atg-5* and *p62* was performed as described previously [35]. Briefly, Atg-5, p62, or negative control small interfering RNA (siRNA) was transfected into cells using the Lipofectamine RNAiMAX Reagent. The knockdown efficiency of these siRNAs was validated by RT-PCR (Fig. S9). RNA preparation, cDNA synthesis, and real time PCR were carried out as described [37], using primer sets (Atg-5; 5′-TTGACGTTGGTAACTGACAAAGT-3′/5′-TGTGATGTTCCAAGGAAGAGC-3′ or p62; 5′-GCTTCTGGTCCATCGG-3′/5′-CGCCCTGAGGAACAG-3′).

## Supporting Information

Figure S1 **Introduction of monomeric α-synuclein.** Monomeric α-synuclein was introduced into HEK293 cells. After 4 h, cells were stained with anti-phosphorylated α-synuclein (P-αSyn), anti-ubiquitin (Ub), and p62 antibodies. Blue, DAPI. Scale bar, 10 µm.
(TIF)

Figure S2 **Induction of autophagy by α-synuclein fibril introduction.** α-Synuclein fibrils were introduced into HEK293 cells stably expressing GFP-LC3 (+Fibrils). After 1 h, cells were stained with anti-phosphorylated α-synuclein antibody (P-αSyn) and observed with a fluorescence microscope. GFP-LC3-positive autophagosomes are indicated by arrowheads. Lower panels are mock-introduced cells (Control). Blue, DAPI. Scale bar, 10 µm.
(TIF)

Figure S3 **Colocalization of LC3, p62, or ubiquitin with phosphorylated α-synuclein-positive inclusions in SH-SY5Y.** α-Synuclein fibrils were introduced into human neuroblastoma SH-SY5Y cells. After 4 h, cells were stained with anti-phosphorylated α-synuclein (P-αSyn), anti-LC3 (LC3; left panel), anti-p62 (p62; middle panel), and anti-ubiquitin (Ub; right panel) antibodies. Images were obtained by a confocal laser microscope. Blue, DAPI. Scale bar, 10 µm.
(TIF)

Figure S4 **Autophagic clearance of α-synuclein inclusions.** HEK293 cells were transfected with negative control (left panels), Atg-5 (middle panels), or p62 (right panels) siRNA. α-Synuclein fibrils were introduced into these cells after 3 days, followed by immunostaining with anti-phosphorylated α-synuclein (green) at 4 h or 24 h after fibril-introduction. Blue, DAPI. Scale bar, 50 µm.
(TIF)

Figure S5 **Recruitment of EGFP-parkin and mitophagy.** HEK293 cells stably co-expressing DsRed-LC3 (red) and EGFP-parkin (green) were treated with 10 µM CCCP (**A**) or DMSO (**B**) for 4 h. Mitochondrial localization was examined by anti-Tom20 antibody (aqua). After CCCP-treatment, EGFP-parkin was colocalized to clustered mitochondria, and DsRed-LC3 puncta were also detected in and around mitochondrial clusters (arrows). Scale bar, 10 µm.
(TIF)

Figure S6 **Sequestration of impaired mitochondria in HEK293 cells.** Sequestration of mitochondria into autophagosomes was observed in the cells harboring α-synuclein inclusions. α-Synuclein fibrils (+Fibrils) were introduced into HEK293-cells co-expressing DsRed-LC3 and EGFP-parkin, followed by 10 µM CCCP-treatment for 4 h. Recruitment of EGFP-parkin to the damaged mitochondria and sequestration of both mitochondria (arrowheads) and α-synuclein inclusions (arrows) into autophagosomes were observed in a single cell. Scale bars, 10 µm. High-magnification views of the boxed area are shown in the lower panels. Mock-introduced cells (-Fibrils) were shown in the upper right panel.
(TIF)

Figure S7 **Mitochondrial expression in HEK293 cells.** Mitochondrial clearance was confirmed in mock- (upper panels) or α-synuclein fibrils-introduced cells (lower panels). After introduction, cells were treated with DMSO (left) or 10 µM CCCP for 4 h (middle) or 16 h (right), and were examined immunocytochemically with anti-Tom20 (red) and anti-phosphorylated α-synuclein antibody (P-αSyn; aqua). Figures were presented as merged images. Scale bar, 10 µm. As compared with Fig. 6B, EGFP-parkin overexpression is required for the accelerated clearance of impaired mitochondria in HEK293 cells.
(TIF)

Figure S8 **Mitochondrial clearance in HEK293 cells expressing EGFP-parkin.** Upper and lower panels in Fig. 6B were separated into individual images. Mock- (**A**) or α-synuclein fibrils-introduced cells (**B**) are shown respectively. Outlines demarcate the edges of cells expressing EGFP-Parkin.
(TIF)

Figure S9 **Validation of the knockdown efficiency.** Atg-5, p62, or negative control siRNA were transfected into HEK293 cells stably expressing GFP-LC3. After 5 days, total RNAs were prepared from these cells, and the expression of Atg-5 or p62 was estimated by real time PCR. Relative level of Atg-5 (**A**) or p62 (**B**) was represented by mean ± s.d. as a graph. Statistical analysis was performed with *t*-test. *p<0.01. This experiment was repeated three times.
(TIF)
