# Supplementary figures and images for: p62/SQSTM1-Dependent Autophagy of Lewy Body-Like α-Synuclein Inclusions
Source: PLoS One. 2012 Dec 31;7(12):e52868. doi: 10.1371/journal.pone.0052868 (PMC3534125; doi:10.1371/journal.pone.0052868)

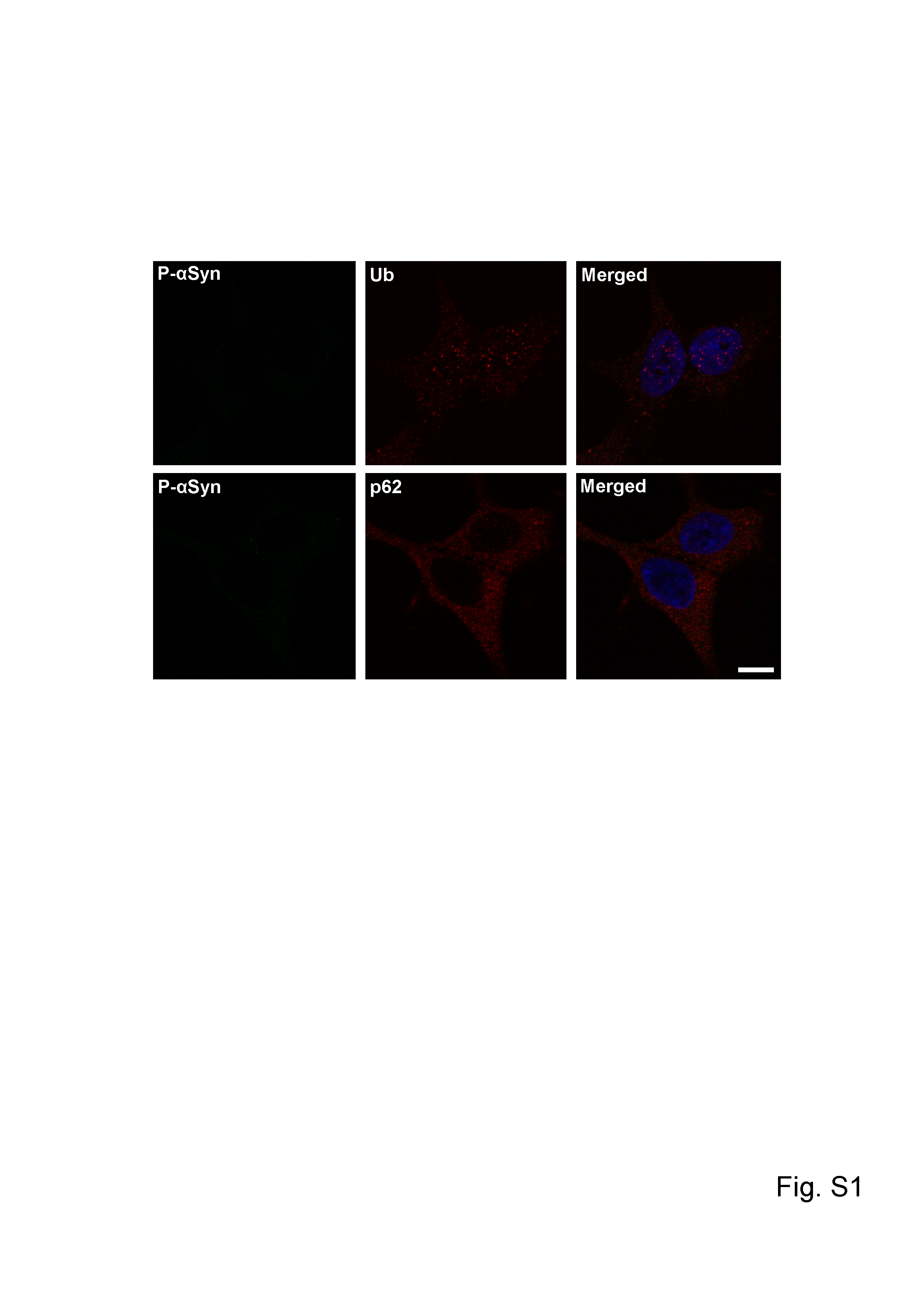

Supplement: Figure S1 — Introduction of monomeric α-synuclein. Monomeric α-synuclein was introduced into HEK293 cells. After 4 h, cells were stained with anti-phosphorylated α-synuclein (P-αSyn), anti-ubiquitin (Ub), and p62 antibodies. Blue, DAPI. Scale bar, 10 µm. (TIF) [file pone.0052868.s001.tif]

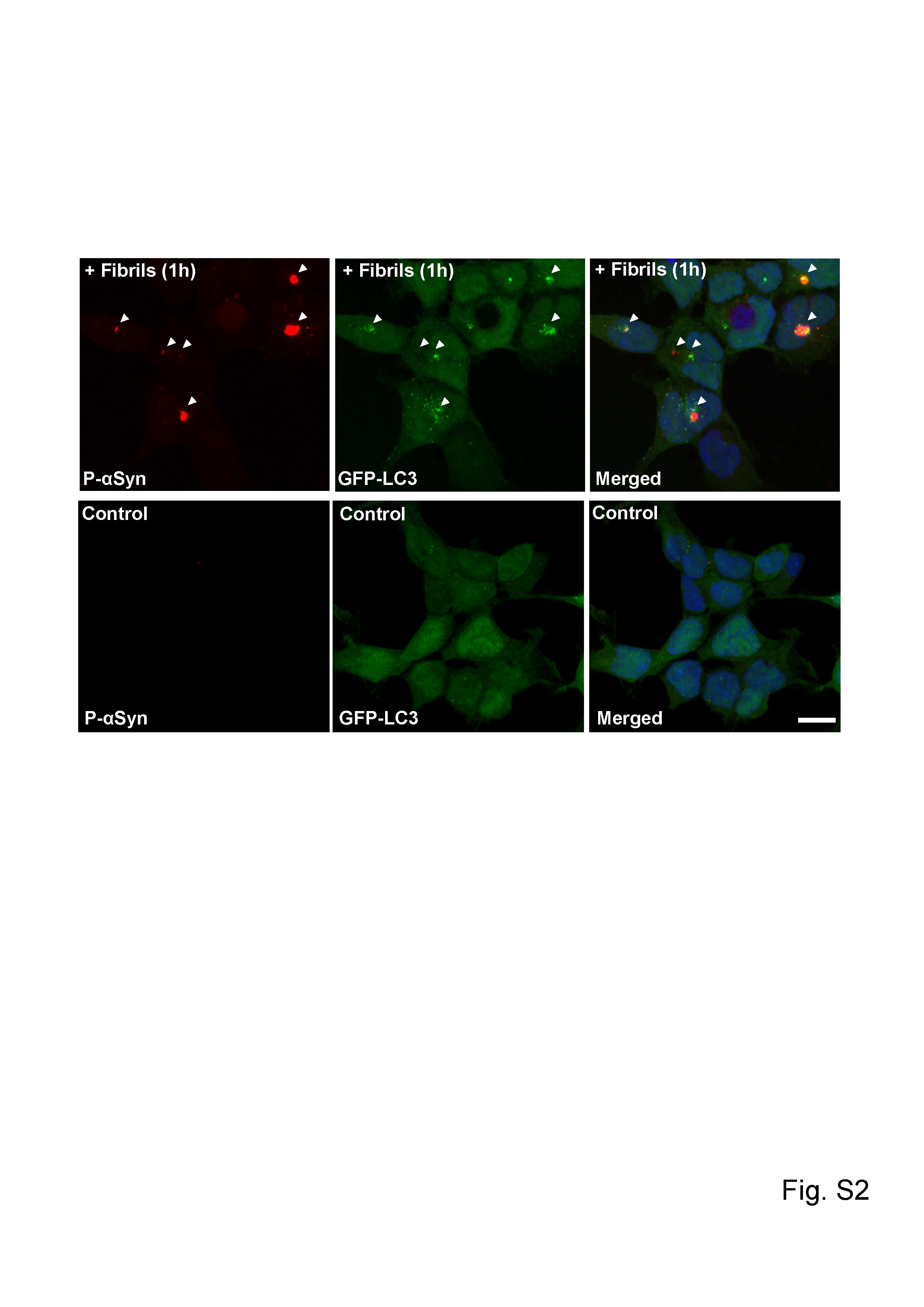

Supplement: Figure S2 — Induction of autophagy by α-synuclein fibril introduction. α-Synuclein fibrils were introduced into HEK293 cells stably expressing GFP-LC3 (+Fibrils). After 1 h, cells were stained with anti-phosphorylated α-synuclein antibody (P-αSyn) and observed with a fluorescence microscope. GFP-LC3-positive autophagosomes are indicated by arrowheads. Lower panels are mock-introduced cells (Control). Blue, DAPI. Scale bar, 10 µm. (TIF) [file pone.0052868.s002.tif]

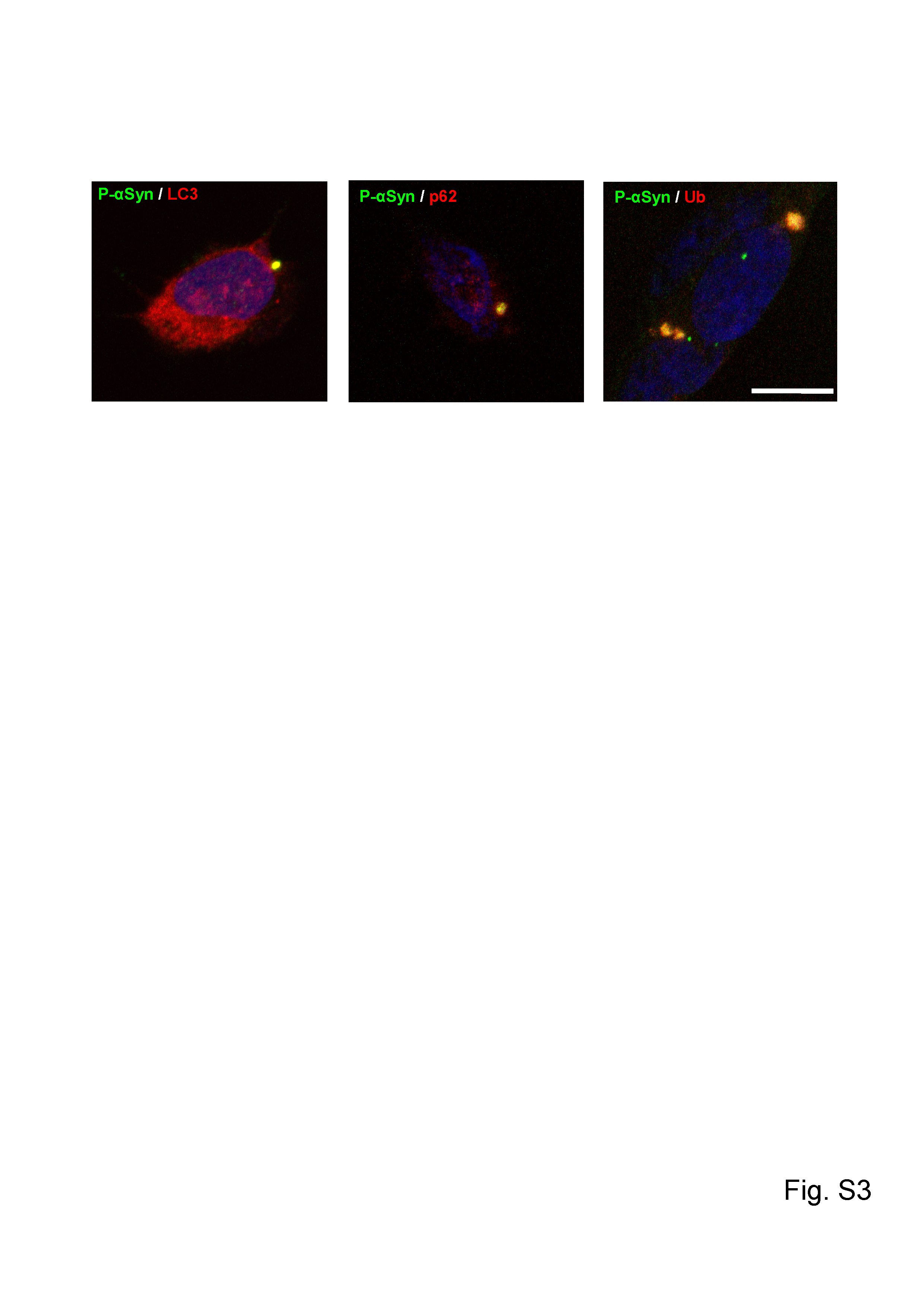

Supplement: Figure S3 — Colocalization of LC3, p62, or ubiquitin with phosphorylated α-synuclein-positive inclusions in SH-SY5Y. α-Synuclein fibrils were introduced into human neuroblastoma SH-SY5Y cells. After 4 h, cells were stained with anti-phosphorylated α-synuclein (P-αSyn), anti-LC3 (LC3; left panel), anti-p62 (p62; middle panel), and anti-ubiquitin (Ub; right panel) antibodies. Images were obtained by a confocal laser microscope. Blue, DAPI. Scale bar, 10 µm. (TIF) [file pone.0052868.s003.tif]

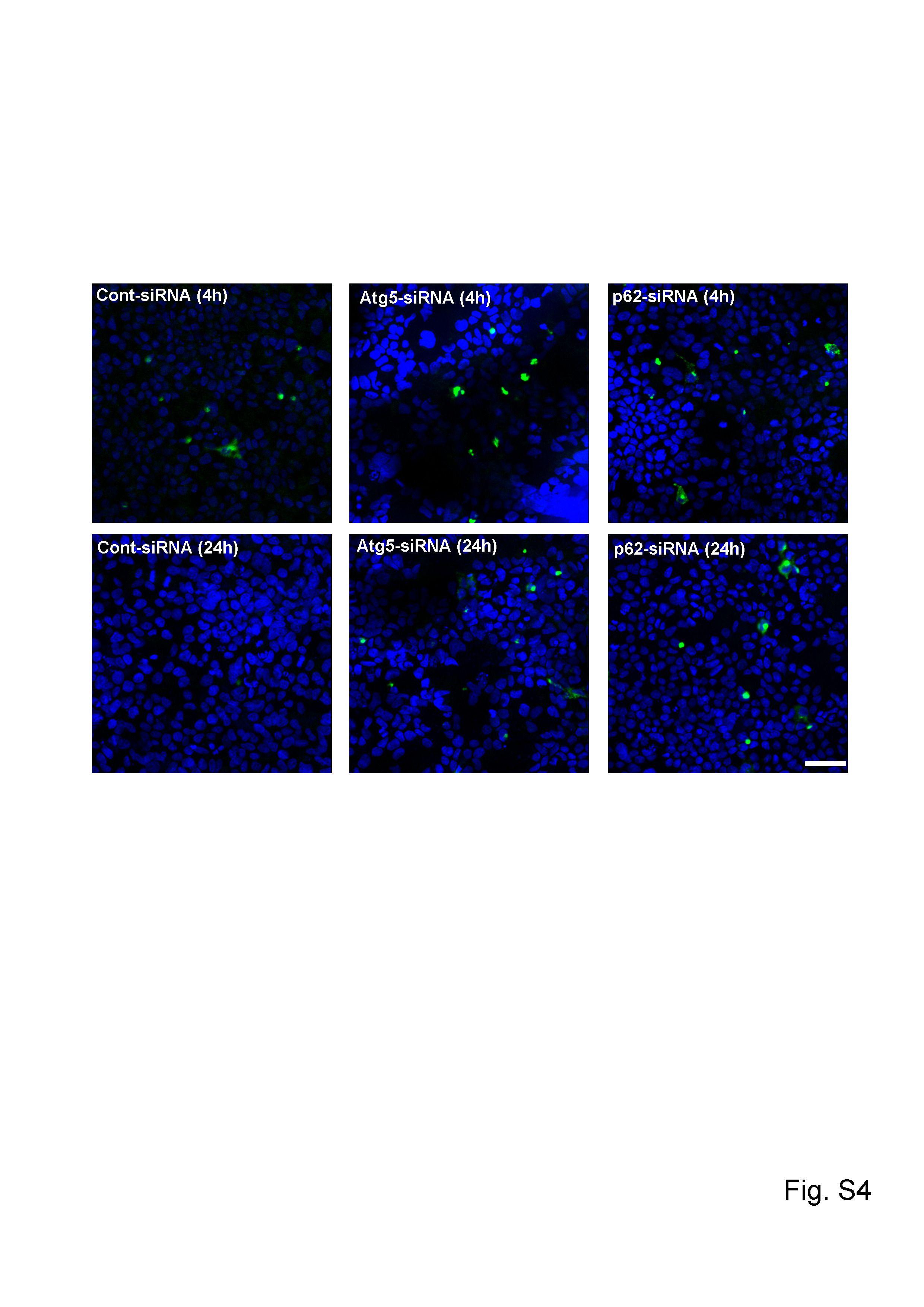

Supplement: Figure S4 — Autophagic clearance of α-synuclein inclusions. HEK293 cells were transfected with negative control (left panels), Atg-5 (middle panels), or p62 (right panels) siRNA. α-Synuclein fibrils were introduced into these cells after 3 days, followed by immunostaining with anti-phosphorylated α-synuclein (green) at 4 h or 24 h after fibril-introduction. Blue, DAPI. Scale bar, 50 µm. (TIF) [file pone.0052868.s004.tif]

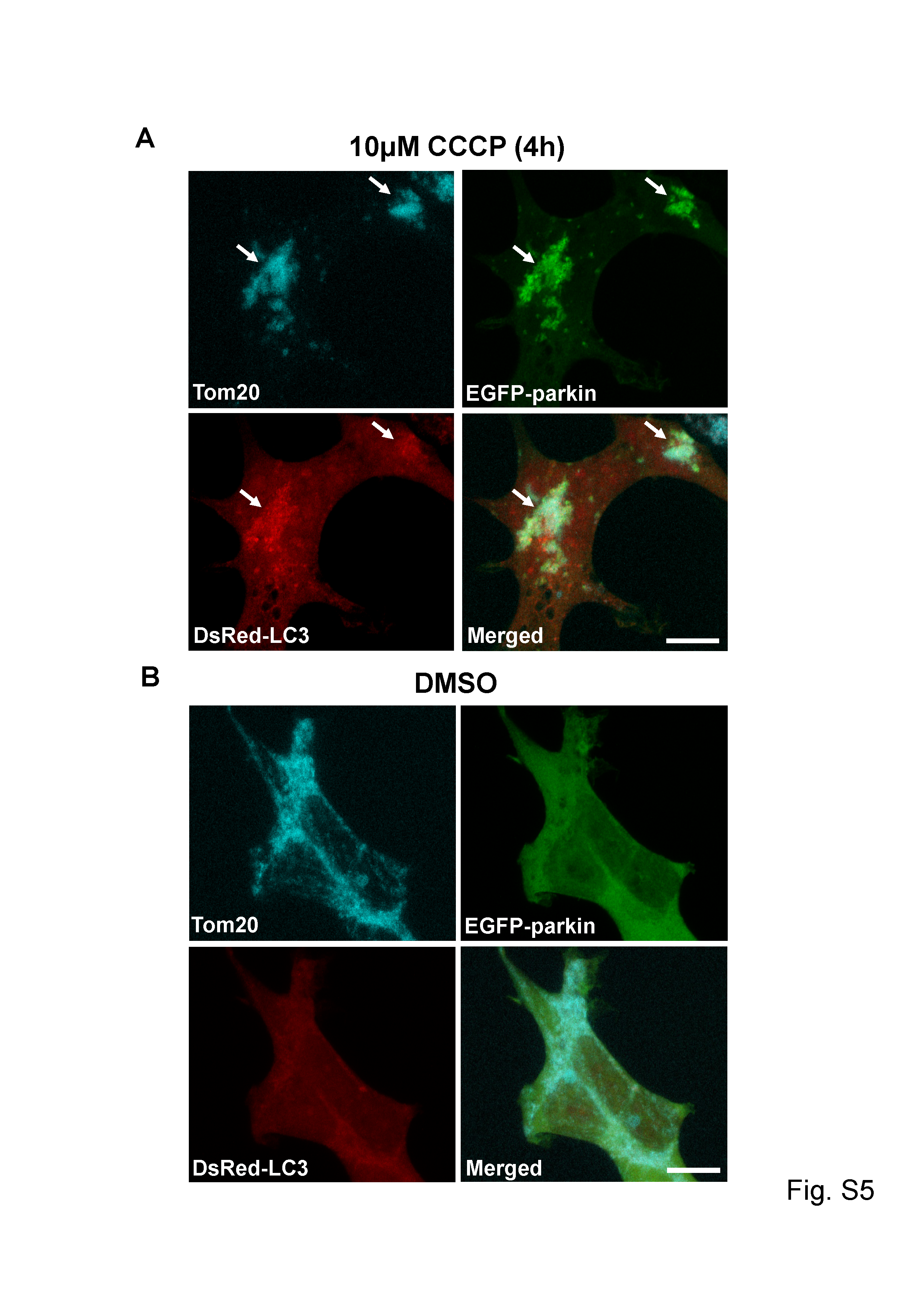

Supplement: Figure S5 — Recruitment of EGFP-parkin and mitophagy. HEK293 cells stably co-expressing DsRed-LC3 (red) and EGFP-parkin (green) were treated with 10 µM CCCP (A) or DMSO (B) for 4 h. Mitochondrial localization was examined by anti-Tom20 antibody (aqua). After CCCP-treatment, EGFP-parkin was colocalized to clustered mitochondria, and DsRed-LC3 puncta were also detected in and around mitochondrial clusters (arrows). Scale bar, 10 µm. (TIF) [file pone.0052868.s005.tif]

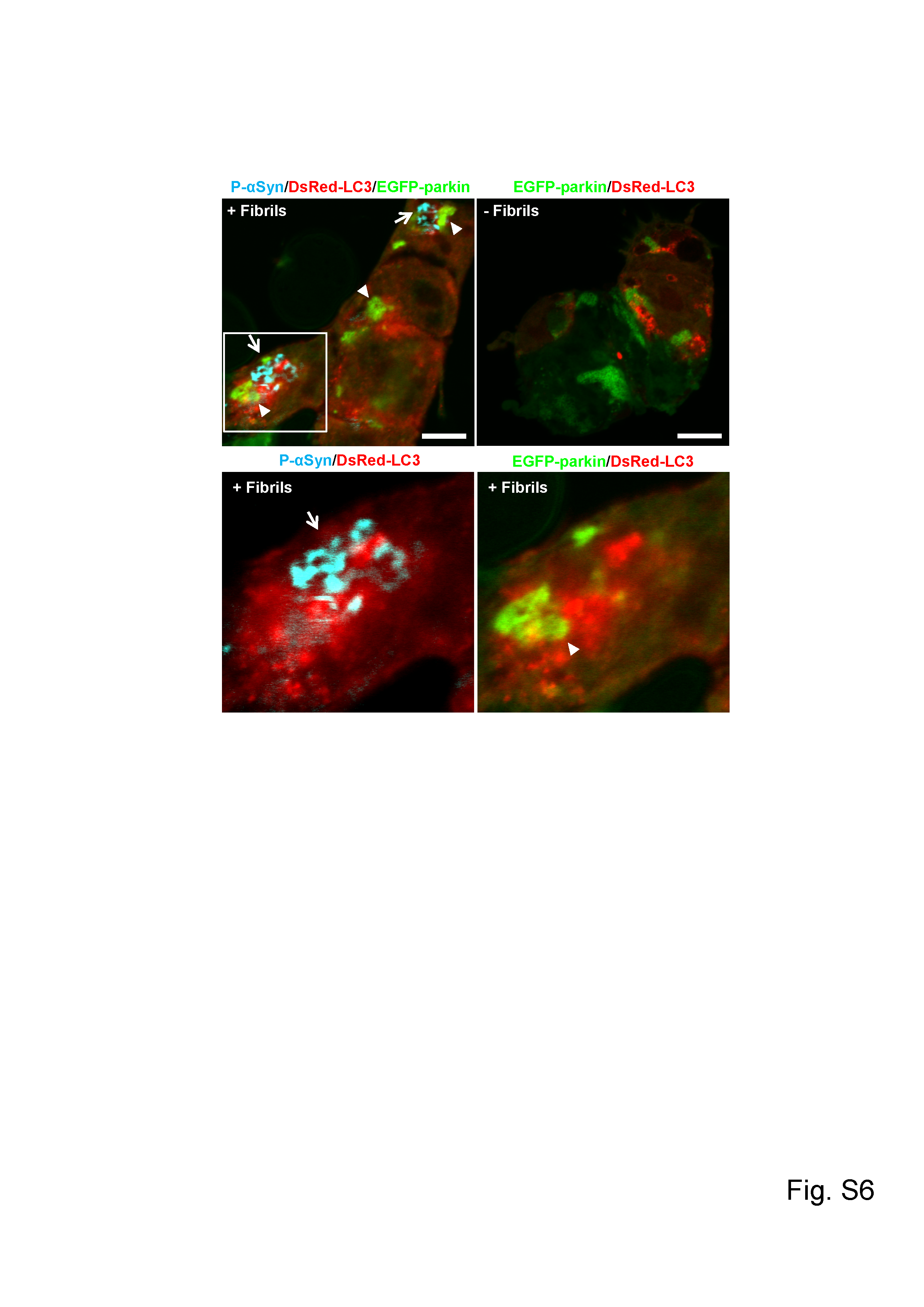

Supplement: Figure S6 — Sequestration of impaired mitochondria in HEK293 cells. Sequestration of mitochondria into autophagosomes was observed in the cells harboring α-synuclein inclusions. α-Synuclein fibrils (+Fibrils) were introduced into HEK293-cells co-expressing DsRed-LC3 and EGFP-parkin, followed by 10 µM CCCP-treatment for 4 h. Recruitment of EGFP-parkin to the damaged mitochondria and sequestration of both mitochondria (arrowheads) and α-synuclein inclusions (arrows) into autophagosomes were observed in a single cell. Scale bars, 10 µm. High-magnification views of the boxed area are shown in the lower panels. Mock-introduced cells (-Fibrils) were shown in the upper right panel. (TIF) [file pone.0052868.s006.tif]

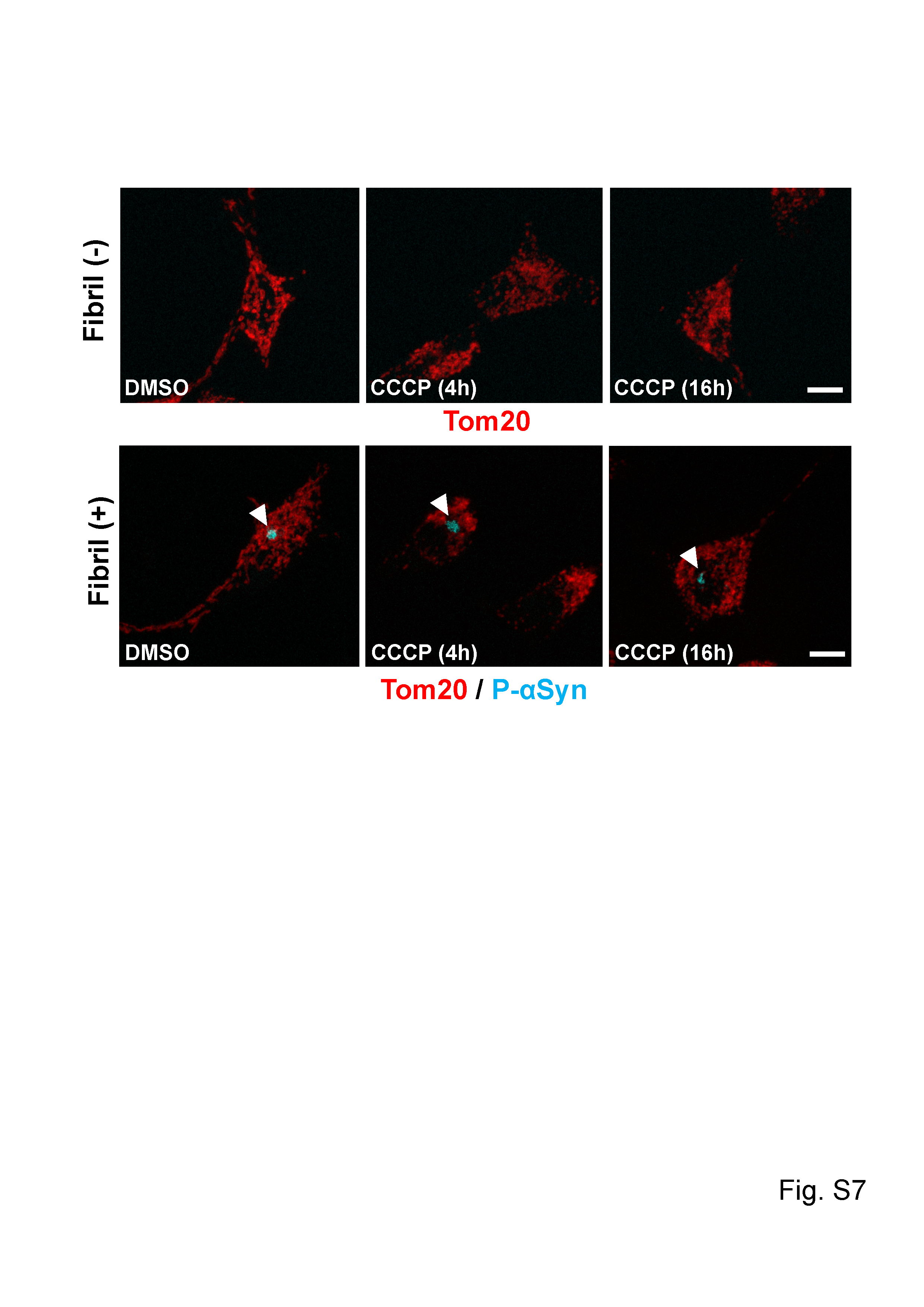

Supplement: Figure S7 — Mitochondrial expression in HEK293 cells. Mitochondrial clearance was confirmed in mock- (upper panels) or α-synuclein fibrils-introduced cells (lower panels). After introduction, cells were treated with DMSO (left) or 10 µM CCCP for 4 h (middle) or 16 h (right), and were examined immunocytochemically with anti-Tom20 (red) and anti-phosphorylated α-synuclein antibody (P-αSyn; aqua). Figures were presented as merged images. Scale bar, 10 µm. As compared with Fig. 6B, EGFP-parkin overexpression is required for the accelerated clearance of impaired mitochondria in HEK293 cells. (TIF) [file pone.0052868.s007.tif]

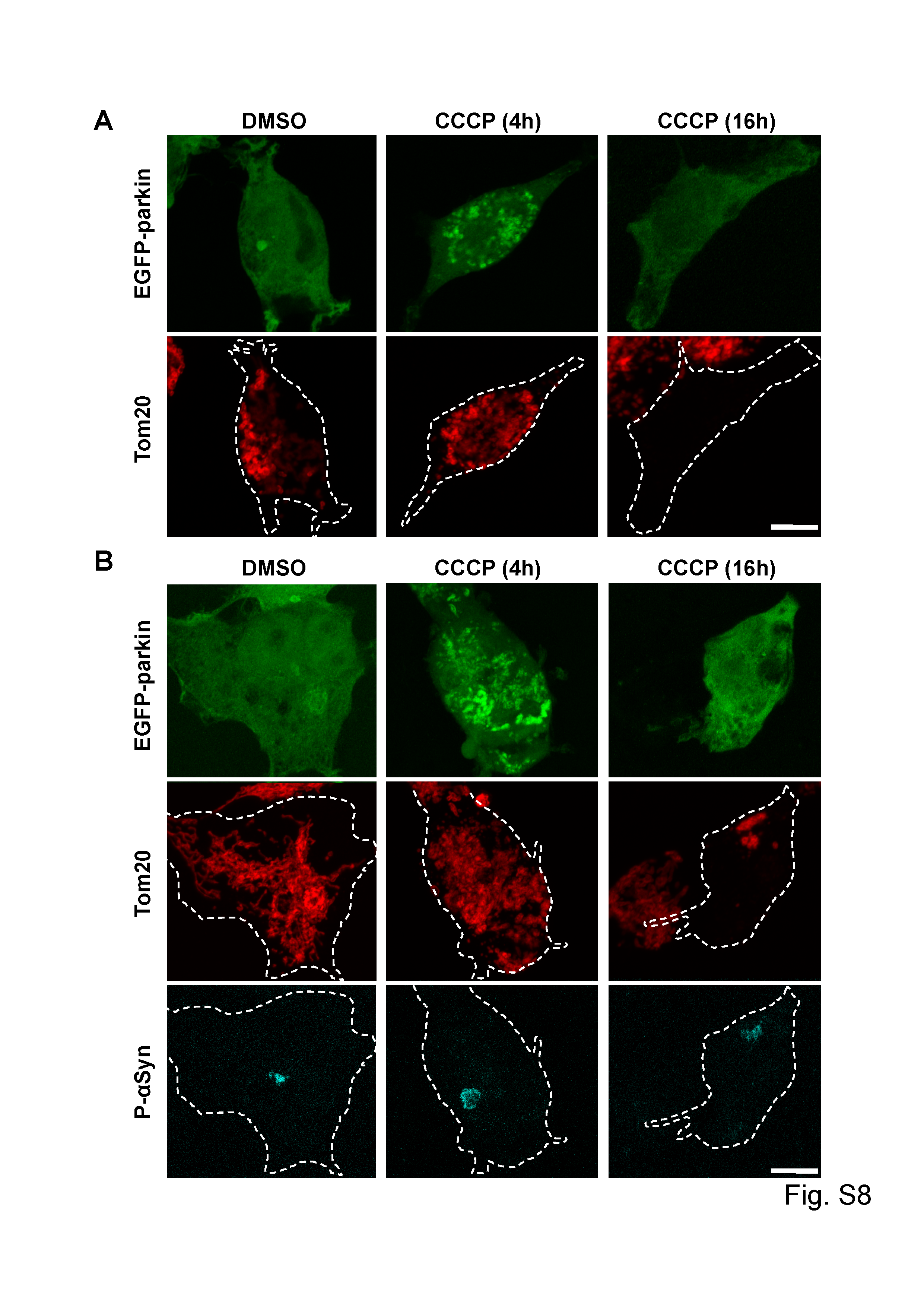

Supplement: Figure S8 — Mitochondrial clearance in HEK293 cells expressing EGFP-parkin. Upper and lower panels in Fig. 6B were separated into individual images. Mock- (A) or α-synuclein fibrils-introduced cells (B) are shown respectively. Outlines demarcate the edges of cells expressing EGFP-Parkin. (TIF) [file pone.0052868.s008.tif]
